# Hobby engagement and mental wellbeing among people aged 65 years and older in 16 countries

**DOI:** 10.1038/s41591-023-02506-1

**Published:** 2023-09-11

**Authors:** Hei Wan Mak, Taiji Noguchi, Jessica K. Bone, Jacques Wels, Qian Gao, Katsunori Kondo, Tami Saito, Daisy Fancourt

**Affiliations:** 1 Department of Behavioural Science and Health, Institute of Epidemiology & Health Care, University College London, London, UK; 2 Department of Social Science, Center for Gerontology and Social Science, Research Institute, National Center for Geriatrics and Gerontology, Obu, Japan; 3 Japan Society for the Promotion of Science, Tokyo, Japan; 4 MRC Unit for Lifelong Health and Ageing, University College London, London, UK; 5 Centre Metices, Université libre Bruxelles, Brussels, Belgium; 6 Department of Social Preventive Medical Sciences, Center for Preventive Medical Sciences, Chiba University, Chiba, Japan; 7 Department of Gerontological Evaluation, Center for Gerontology and Social Science, Research Institute, National Center for Geriatrics and Gerontology, Obu, Japan

**Keywords:** Quality of life, Epidemiology

## Abstract

Growing aging populations pose a threat to global health because of the social and psychological challenges they experience. To mitigate this, many countries promote hobby engagement to support and improve mental health. Yet, it remains unclear whether there is consistency in benefits across different national settings. We harmonized measures of hobby engagement and multiple aspects of mental wellbeing across 16 nations represented in five longitudinal studies (*N* = 93,263). Prevalence of hobby engagement varied substantially across countries, from 51.0% of Spanish respondents to 96.0% of Danish respondents. Fixed effects models and multinational meta-analyses were applied to compare the longitudinal associations between hobbies and mental wellbeing. Independent of confounders, having a hobby was associated with fewer depressive symptoms (pooled coefficient = −0.10; 95% confidence intervals (CI) = −0.13, −0.07), and higher levels of self-reported health (pooled coefficient = 0.06; 95% CI = 0.03, 0.08), happiness (pooled coefficient = 0.09; 95% CI = 0.06, 0.13) and life satisfaction (pooled coefficient = 0.10; 95% CI = 0.08, 0.12). Further analyses suggested a temporal relationship. The strength of these associations, and prevalence of hobby engagement, were correlated with macrolevel factors such as life expectancy and national happiness levels but overall, little variance in findings was explained by country-level factors (<9%). Given the relative universality of findings, ensuring equality in hobby engagement within and between countries should be a priority for promoting healthy aging.

## Abstract (web summary)

A meta-analysis of longitudinal studies representing 16 countries found that hobby engagement was associated with better mental and self-reported health in adults aged 65 years and older as well as correlation with higher life expectancy and national happiness levels.

## Main

Aging populations are an increasing global concern given the social and psychological challenges they can experience, including loneliness, social isolation and worsening mental health, all of which are associated with increasing physical multimorbidity and mortality^1,2^. Globally, the population aged 65 years and older (65+) is growing at a faster rate than all other age groups^3^. According to data from the United Nations, 1 in 11 people were aged 65+ in 2019, which is expected to rise to 1 in 6 people by 2050 (ref. ^3^). Although advancements in healthcare have helped people live longer, healthy life expectancy (the average number of years that a person is expected to live with good health and without any disability, physical or psychological illnesses or injuries) is often not matched with the increase in life expectancy, and there is a growing prevalence of long-term mental health conditions. This is placing untenable burdens on global health and social care services, providing financial and workforce planning predicaments. To help meet older adults’ needs and to support the sustainability of health and social care systems globally, it is important to explore cost-effective strategies to enhance older adults’ mental health and wellbeing.

There is increasingly global interest in how engagement in psychosocial activities could address these challenges^4,5^. Hobbies (defined as activities that people engage in during their leisure time for pleasure, such as the arts, crafts, reading, playing games, sports, gardening, volunteering and participating in societies/clubs) involve imagination, novelty, creativity, sensory activation, self-expression, relaxation and cognitive stimulation, all of which are positively related to mental health and wellbeing via psychological, biological, social and behavioral pathways^5^. Participation in hobby groups can additionally provide social support and reduce loneliness and social isolation^5^. For this reason, many countries including the UK^6^, Japan^7^ and the USA^8^ have been promoting hobbies and leisure activities as part of their policies and recommendations to support and improve mental health and wellbeing, with a particular focus on increasing participation among older adults.

These policies are underpinned by a large body of research that has shown how hobbies can enhance multidimensional aspects of mental health and wellbeing, including negative symptomatology and clinical diagnoses of depression and psychiatric conditions, experiential wellbeing (for example, positive and negative affect), evaluative wellbeing (for example, life satisfaction) and eudemonic wellbeing (for example, purpose in life) for older adults. Meta-analyses of both observational and interventional studies involving engagement in hobbies such as nature-based activities and volunteering have shown protective associations with depressive symptoms^9–12^. These findings are supported by individual studies showing concurrent and longitudinal relationships (3–12 years of follow-up) between other types of hobbies such as community groups, arts and social clubs, and a lower incidence and prevalence of depression in adults aged 50 years and older (50+) in the USA^13^, Japan^14^, the UK^15^ and China^16^. Similarly, meta-analyses of various types of leisure activities, such as dancing, nature-based activities and gardening, have reported benefits for positive aspects of wellbeing^10,17–20^. Again, these findings are supported by individual studies focusing on broader activities such as volunteering, arts, cultural engagement and indoor gardening from Sweden^21^, the UK^22^, Japan^23^ and the USA^24^.

However, the literature to date is hampered by several limitations. First, studies have focused on single countries at a time, so given differences in definitions, outcome measures and methodological approaches between studies, it is unclear whether there is consistency in results across different cultural settings, and thus whether findings from one country population could be applied to populations in other countries. Second, many studies have focused on specific subcategories of hobbies (for example, volunteering versus nature-based activities versus arts participation versus cultural engagement), often applying conflicting definitions. Yet all hobbies share common ‘active ingredients’ and activate similar causal mechanisms of action; it has been proposed that there is little to differentiate in their potential to affect population-level mental health outcomes^25,26^. Individual meta-analyses focusing on specific hobby definitions thus present only a fraction of the literature available on the topic and provide an incomplete picture to policymakers.

This study was therefore designed to harmonize measures of hobby engagement and mental wellbeing in adults aged 65+ across 16 nations represented in five longitudinal studies, and explore the relationship with mental wellbeing, the direction of association and the variation in findings by country.

## Results

### Participants

We undertook fixed effect analyses and multinational meta-analyses of longitudinal data from the English Longitudinal Study of Ageing (ELSA, Waves 7–9), Japan Gerontological Evaluation Study (JAGES, Waves 2–4), US Health and Retirement Study (HRS, Waves 9–14), Survey of Health, Ageing and Retirement in Europe (SHARE, Waves 4–6) and China Health and Retirement Longitudinal Study (CHARLS, Waves 1–3). ELSA, JAGES, HRS and CHARLS follow participants living in England, Japan, the USA and China, respectively. SHARE follows participants living in 28 European countries and Israel but, for this study, we focused only on participants living in Austria, Belgium, Czech Republic, Denmark, Estonia, France, Germany, Italy, Slovenia, Spain, Sweden and Switzerland, where data were available for the analysis. We followed participants for three consecutive waves (4–8 years).

To allow for comparison across all datasets, we limited participants to those aged 65+. To explore how changes in hobby engagement were associated with changes in mental wellbeing over time, a total of 93,263 respondents who provided data across all study measures were analyzed: Austria (*n* = 2,524), Belgium (*n* = 2,304), China (*n* = 1,611), Czech Republic (*n* = 2,664), Denmark (*n* = 1,006), England (*n* = 4,267), Estonia (*n* = 3,584), France (*n* = 2,705), Germany (*n* = 966), Italy (*n* = 1,915), Japan (*n* = 57,051), Slovenia (*n* = 1,272), Spain (*n* = 2,099), Sweden (*n* = 1,315), Switzerland (*n* = 1,776) and the USA (*n* = 6,204).

**Fig. 1:**
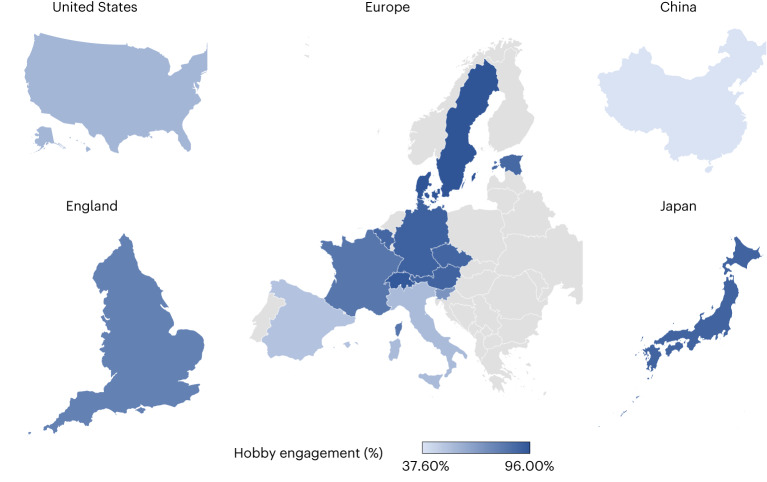
Levels of hobby engagement. Levels of hobby engagement among older adults aged 65 and above across 16 nations.

The average age of the respondents across the different countries was between 71.7 and 75.9 years. Generally, there was a higher proportion of females participating in the surveys (except for China, Japan, and Germany). More than seven out of ten were retired, except for those living in China, Japan and Spain. More than 60% of the participants experienced long-standing mental or physical health conditions (Table 1). For hobby engagement, Denmark (96.0%), Sweden (95.8%) and Switzerland (94.4%) had the highest engagement levels, followed by Germany (91.0%), Austria (90.0%) and Japan (90.0%). Italy (54.0%), Spain (51.0%) and China (37.6%; albeit focusing exclusively on social hobbies) had the lowest engagement levels (Fig. 1 and Table 1).

**Table 1:** Basic demographics by country in percentages or mean (s.d.)

	Austria	Belgium	China	Czech Republic	Denmark	England	Estonia	France	Germany	Italy	Japan	Slovenia	Spain	Sweden	Switzerland	USA
Hobby (%)																
With hobby	90.0	88.9	37.6	89.1	96.0	78.1	88.3	82.5	91.0	54.0	90.0	70.5	51.0	95.8	94.4	56.2
Without hobby	9.1	11.1	62.4	10.9	4.0	21.9	11.7	17.5	9.0	46.0	10.0	29.5	49.0	4.20	5.60	43.8
*n*	2,524	2,304	1,611	2,664	1,006	4,267	3,584	2,705	966	1,915	57,051	1,272	2,099	1,315	1,776	6,204
Gender (%)																
Female	58.1	55.8	45.5	57.6	54.2	53.7	61.6	58.0	48.7	51.4	45.1	56.6	54.3	52.8	53.0	58.4
Male	41.9	44.2	54.5	42.4	45.8	46.3	38.4	42.0	51.3	48.6	54.9	43.4	45.7	47.2	47.0	41.6
*n*	2,524	2,304	1,611	2,664	1,006	4,267	3,584	2,705	966	1,915	57,051	1,272	2,099	1,315	1,776	6,204
Age (s.d.)	74.2 (6.53)	75.5 (7.25)	71.7 (4.98)	73.5 (6.52)	74.8 (7.42)	73.8 (6.65)	74.6 (6.16)	75.8 (7.17)	74.0 (6.33)	74.1 (6.46)	73.5 (5.60)	74.7 (6.41)	75.9 (7.16)	74.7 (7.22)	74.2 (6.82)	72.6 (6.06)
Range	65–98	65–101	65–92	65–98.9	65–99	65–99	65–101	65–103	65–100	65–100	65–99	65–99	65–101	65–99	65–101	65–101
*n*	2,524	2,304	1,611	2,664	1,006	4,267	3,584	2,705	966	1,915	57,051	1,272	2,099	1,315	1,776	6,204
Employment status (%)																
Working	1.5	1.0	42.9	1.0	4.3	11.2	9.6	0.9	3.2	1.6	27.6	0.5	1.0	4.4	5.2	14.6
Not working	13.5	16.3	0.9	0.2	3.2	4.27	1.2	7.1	6.4	23.1	6.5	10.5	37.6	0.2	8.6	7.0
Retired	85.0	82.7	56.2	98.8	92.5	84.5	89.2	92.0	90.4	75.3	66.0	89.0	61.4	95.4	86.2	78.3
*n*	2,458	2,216	1,611	2,612	973	4,267	3,568	2,620	950	1,906	57,051	1,259	2,073	1,293	1,742	6,204
Long-standing mental/physical health conditions (%)																
Yes	73.7	77.0	84.1	83.8	68.4	60.2	83.0	75.9	75.1	74.9	81.4	76.7	78.0	67.3	66.1	93.6
No	26.3	23.0	15.9	16.2	31.6	39.8	17.0	24.1	24.9	25.1	18.6	23.3	22.0	32.7	33.9	6.4
*n*	2,504	2,295	1,611	2,643	1,004	4,267	3,576	2,665	966	1,912	57,051	1,260	2,091	1,313	1,770	6,204

Note: The table shows baseline demographics where baseline indicates the first wave at which each participant completed the survey, and therefore does not relate to a single year of data.

### Longitudinal associations between hobby engagement and mental wellbeing

Fixed effects models tested the longitudinal associations of how changes in engagement in hobbies were associated with changes in mental wellbeing, simultaneously accounting for all time-constant factors (regardless of whether they were observed; for example, genetics, past leisure behaviors, medical histories and psychological traits) and identified time-varying factors (for example, sociodemographic backgrounds, clinical conditions and difficulties with activities of daily living). We then pooled our findings into novel multinational meta-analyses.

**Fig. 2:**
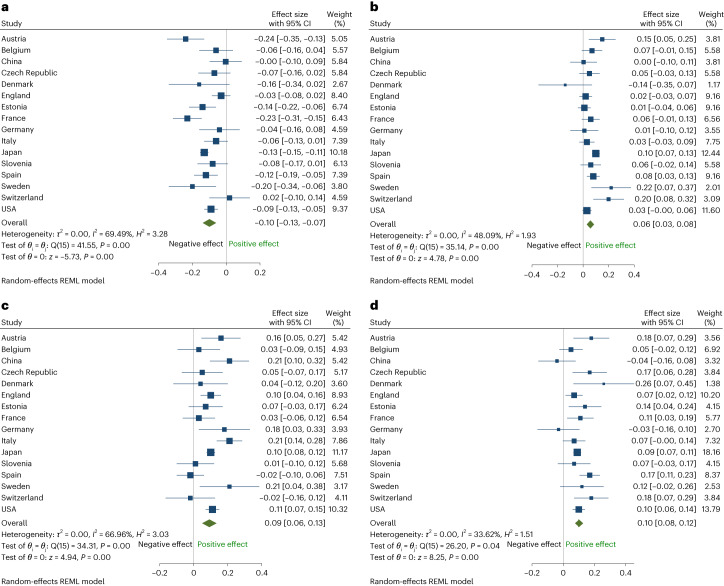
Meta-analysis of the findings from fixed effects models (*n* study = 16). Data were first analyzed separately for each country using fixed effects regression. The findings were then pooled into multinational meta-analyses using the random effects model to estimate the overall effect sizes for all outcomes. Between-study heterogeneity was estimated using the algorithm of the restricted maximum likelihood and was assessed using *I*^2^ and *H*^2^ statistics. *I*^2^ is the percentage of variability in the effect size that is caused by between-study heterogeneity, rather than by sampling error. The *H*^2^ statistic describes the ratio of the observed variation and the expected variance due to sampling error. Given that some of the analyses had more participants than others and thus had lower sampling variability and more precise estimates, the meta-analysis was weighted. Studies with a greater number of respondents were given more weight than studies with a small number of respondents. These were relative weights that summed to 100. Data are presented as fixed effects coefficients and 95% CI. The overall effect size and its width should have accounted for the between-study variance, the number of studies, the precision of the study-specific estimates (or ‘effect sizes’) and the significance level. **a**, Hobbies and depressive symptoms. **b**, Hobbies and self-reported health. **c**, Hobbies and happiness. **d**, Hobbies and life satisfaction.

Overall, hobby engagement was negatively associated with depressive symptoms (pooled coefficient = −0.10; 95% CI = −0.13, −0.07; *I*^2^ = 69.5%; *H*^2^ = 3.28; where *I*^2^ is the percentage of variability in the effect size that is caused by between-study heterogeneity, rather than by sampling error, and the *H*^2^ statistic describes the ratio of the observed variation and the expected variance due to sampling error), positively associated with self-reported health (pooled coefficient = 0.06; 95% CI = 0.03, 0.08; *I*^2^ = 48.1%; *H*^2^ = 1.93), positively associated with happiness (pooled coefficient = 0.09; 95% CI = 0.06, 0.13; *I*^2^ = 67.0%; *H*^2^ = 3.03), and positively associated with life satisfaction (pooled coefficient = 0.10; 95% CI = 0.08, 0.12; *I*^2^ = 33.6%; *H*^2^ = 1.51) (Fig. 2).

### Directionality

Although fixed effects regression showed the nature of the relationship between hobby and mental wellbeing, the directionality of this relationship required further investigation. So we ran ordinary least squares (OLS) regressions estimating the associations between hobbies measured at Time 1 and the outcomes measured at Time 2, while controlling for identified confounders and baseline outcomes. Results were then pooled into meta-analyses.

**Extended Data Fig. 1:**
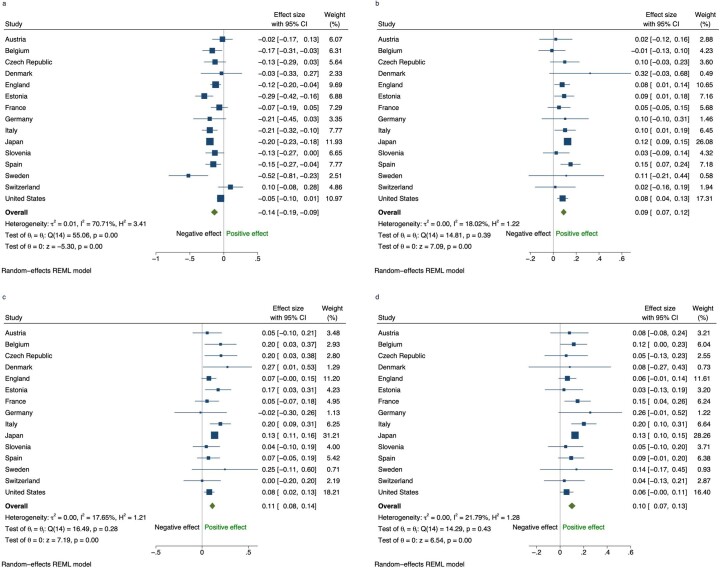
Directionality testing using Ordinary Least Squares (OLS) regressions. N study = 15. Data were first analysed separately for each country using OLS regression. OLS regressions were applied to estimate the association between hobbies measured at Time 1 and outcomes measured at Time 2, while controlling for baseline outcomes measured, age, gender, the number of people living in the household, partnership status, household income, housing tenure, employment status, educational level, long-term mental/physical conditions, difficulties with daily activities (ADLs), and difficulties with instrumental activities of daily living (IADLs). The findings were then pooled into multi-national meta-analyses using the random effect model to estimate the overall effect sizes for all outcomes. Between-study heterogeneity was estimated using the algorithm of the restricted maximum likelihood and was assessed using I^2^ and H^2^ statistics. I^2^ indicates the percentage of variability in the effect size that is caused by between-study heterogeneity, rather than by sampling error. H^2^ statistics describes the ratio of the observed variation and the expected variance due to sampling error. Given that some of the analyses had more participants than others and thus had lower sampling variability and more precise estimates, the meta-analysis was weighted. Studies with a greater number of respondents were given more weight than studies with a small number of respondents. These were relative weights that summed to 100. Data are presented as OLS coefficients and 95% confidence intervals. The overall effect size and its width should have accounted for the between-study variance, the number of studies, the precision of the study-specific estimates (or ‘effect sizes’) and the significance level. **a**. Hobbies and depressive symptoms. **b**. Hobbies and self-reported health. **c**. Hobbies and happiness. **d**. Hobbies and life satisfaction.

Hobby engagement was associated with subsequently fewer depressive symptoms (pooled coefficient = −0.14; 95% CI = −0.19, −0.09; *I*^2^ = 70.7%; *H*^2^ = 3.41) and greater self-reported health (pooled coefficient = 0.09; 95% CI = 0.07, 0.12; *I*^2^ = 18.0%; *H*^2^ = 1.22), happiness (pooled coefficient = 0.11; 95% CI = 0.08, 0.14; *I*^2^ = 17.7%; *H*^2^ = 1.21) and life satisfaction (pooled coefficient = 0.10; 95% CI = 0.07, 0.13; *I*^2^ = 21.8%; *H*^2^ = 1.28) (Extended Data Fig. 1).

We tested the consistency of these findings using a different statistical approach—lagged fixed effects models using an Arellano–Bond estimator model—on the ELSA dataset where there were sufficient repeated waves (nine available). Results confirmed that hobby engagement was still associated with subsequent changes in depressive symptoms (coefficient = −0.38; 95% CI = −0.63, −0.12), self-reported health (coefficient = 0.73; 95% CI = 0.47, 0.99) and happiness (coefficient = 0.36; 95% CI = 0.01, 0.71), with marginal effects on life satisfaction (coefficient = 0.19; 95% CI = −0.03, 0.41) (Supplementary Table 1).

### Country-level factors

**Extended Data Fig. 2:**
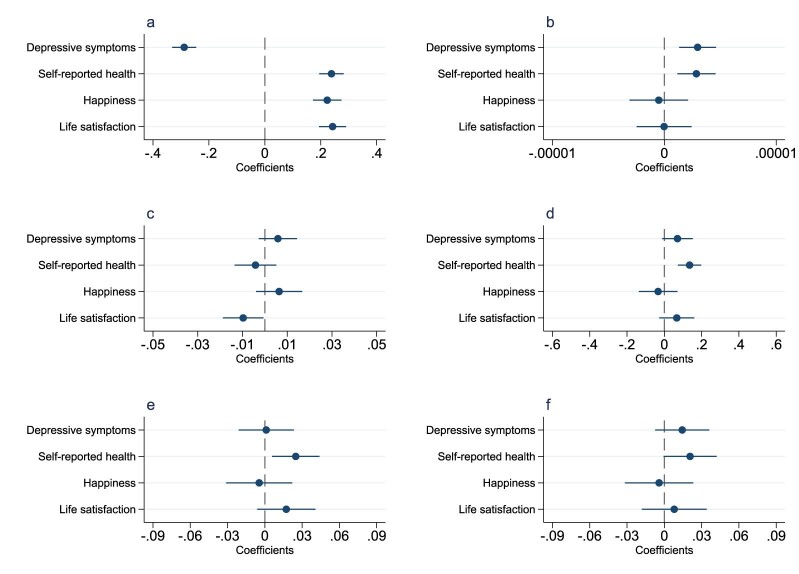
Multi-level models testing the moderating effects of country-level factors. N study = 15. Multi-level analyses were performed. All models controlled for interview waves, type of hobby measure, age, gender, the number of people living in the household, partnership status, household income, housing tenure, employment status, educational level, long-term mental/physical conditions, difficulties with daily activities (ADLs), difficulties with instrumental activities of daily living (IADLs). Data are presented as coefficients and 95% confidence intervals. **a**. No interaction. **b**. Interacting with country wealth. **c**. Interacting with Gini index. **d**. Interacting with world happiness index. **e**. Interacting with life expectancy. **f**. Interacting with national pension age.

To ascertain how much of the variance in the relationship with mental wellbeing was explained by country, we merged the datasets and ran multilevel models. After adjusting for confounders, associations between hobbies and the outcomes remained, and the country variance explained <9% of the total variance (Extended Data Fig. 2).

**Fig. 3:**
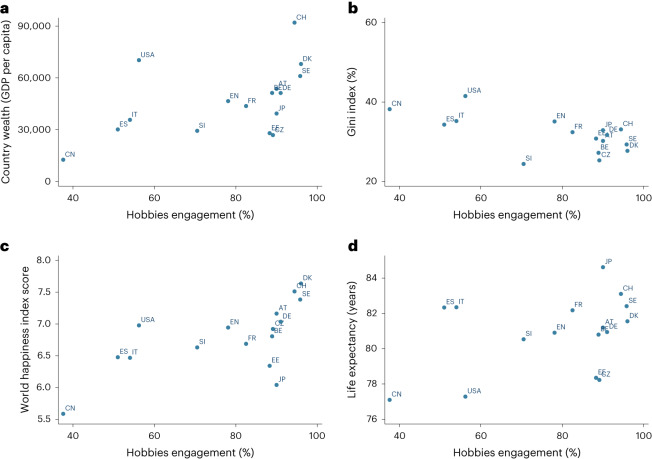
Correlations between hobby engagement rate and country-level factors (*n* study = 16). **a**, Hobbies and country wealth. **b**, Hobbies and Gini index. **c**, Hobbies and world happiness index. **d**, Hobbies and life expectancy. Data are presented as mean values. AT, Austria; BE, Belgium; CN, China; CZ, Czech Republic; DK, Denmark; EN, England; EE, Estonia; FR, France; DE, Germany; IT, Italy; JP, Japan; SI, Slovenia; ES, Spain; SE, Sweden; CH, Switzerland; USA, United States of America.

We then explored which country-level factors might explain this variance. Prevalence of hobby engagement was positively correlated with the world happiness index score^27^ (*r* = 0.63), country wealth measured by gross domestic product by capita^28^ (*r* = 0.49) and life expectancy^29^ (*r* = 0.39), and was negatively correlated with the Gini index measuring income inequality within a nation^30^ (*r* = −0.63) (Fig. 3).

**Extended Data Fig. 3:**
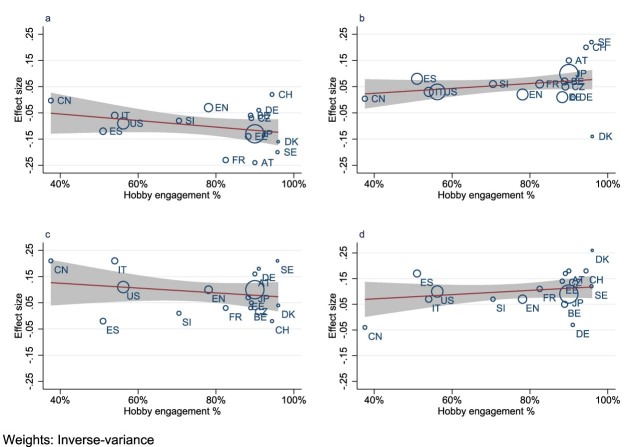
Bubble plot with fitted meta regression line of the effect size for four outcomes and the prevalence of hobby engagement. N study = 16. Data are presented as fixed effects coefficients (bubbles), 95% confidence intervals (shaded area) and the linear prediction (red line). **a**. Depressive symptoms. **b**. Self-reported health. **c**. Happiness. **d**. Life satisfaction. AT=Austria, BE=Belgium, CN=China, CZ=Czech Republic, DK=Denmark, EN=England, EE=Estonia, FR=France, DE=Germany, IT=Italy, JP=Japan, SI=Slovenia, ES=Spain, SE=Sweden, CH=Switzerland, and US=United States.

**Extended Data Fig. 4:**
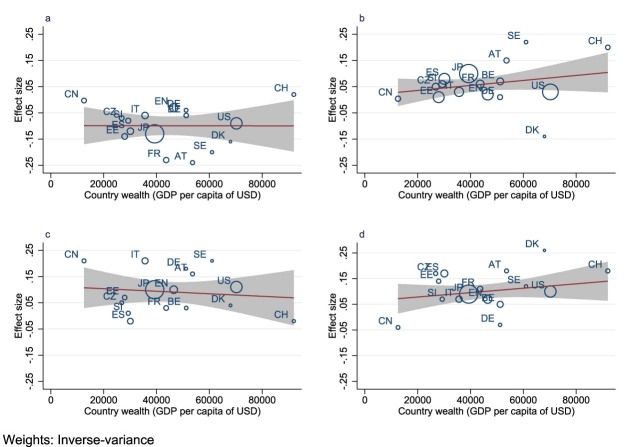
Bubble plot with fitted meta regression line of the effect size for four outcomes and country wealth (GDP per capita of USD). N study = 16. Data are presented as fixed effects coefficients (bubbles), 95% confidence intervals (shaded area) and the linear prediction (red line). **a**. Depressive symptoms. **b**. Self-reported health. **c**. Happiness. **d**. Life satisfaction. AT=Austria, BE=Belgium, CN=China, CZ=Czech Republic, DK=Denmark, EN=England, EE=Estonia, FR=France, DE=Germany, IT=Italy, JP=Japan, SI=Slovenia, ES=Spain, SE=Sweden, CH=Switzerland, and US=United States.

**Extended Data Fig. 5:**
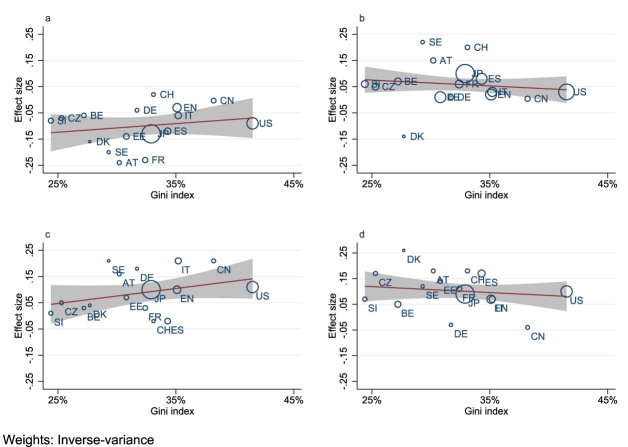
Bubble plot with fitted meta regression line of the effect size for four outcomes and Gini index. N study = 16. Data are presented as fixed effects coefficients (bubbles), 95% confidence intervals (shaded area) and the linear prediction (red line). **a**. Depressive symptoms. **b**. Self-reported health. **c**. Happiness. **d**. Life satisfaction. AT=Austria, BE=Belgium, CN=China, CZ=Czech Republic, DK=Denmark, EN=England, EE=Estonia, FR=France, DE=Germany, IT=Italy, JP=Japan, SI=Slovenia, ES=Spain, SE=Sweden, CH=Switzerland, and US=United States.

**Extended Data Fig. 6:**
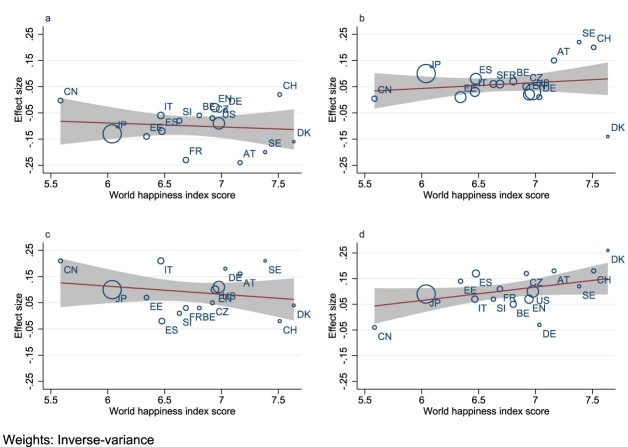
Bubble plot with fitted meta regression line of the effect size for four outcomes and world happiness index. N study = 16. Data are presented as fixed effects coefficients (bubbles), 95% confidence intervals (shaded area) and the linear prediction (red line). **a**. Depressive symptoms. **b**. Self-reported health. **c**. Happiness. **d**. Life satisfaction. AT=Austria, BE=Belgium, CN=China, CZ=Czech Republic, DK=Denmark, EN=England, EE=Estonia, FR=France, DE=Germany, IT=Italy, JP=Japan, SI=Slovenia, ES=Spain, SE=Sweden, CH=Switzerland, and US=United States.

**Extended Data Fig. 7:**
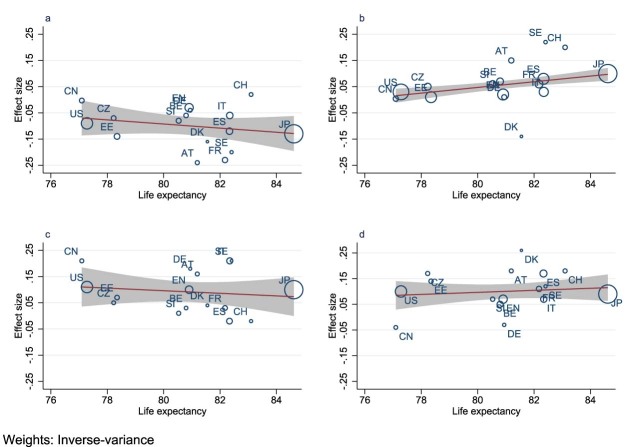
Bubble plot with fitted meta regression line of the effect size for four outcomes and life expectancy. N study = 16. Data are presented as fixed effects coefficients (bubbles), 95% confidence intervals (shaded area) and the linear prediction (red line). **a**. Depressive symptoms. **b**. Self-reported health. **c**. Happiness. **d**. Life satisfaction. AT=Austria, BE=Belgium, CN=China, CZ=Czech Republic, DK=Denmark, EN=England, EE=Estonia, FR=France, DE=Germany, IT=Italy, JP=Japan, SI=Slovenia, ES=Spain, SE=Sweden, CH=Switzerland, and US=United States.

These same country-level factors were also used in meta-regressions as potential predictors of between-study heterogeneity in outcomes. For the prevalence of hobby engagement (Extended Data Fig. 3), country wealth (Extended Data Fig. 4) and Gini index (Extended Data Fig. 5), no differences in effect sizes were found according to these predictors. For the world happiness index score, no associations were shown between effect sizes and index score, except marginally for life satisfaction (Extended Data Fig. 6). With a confidence level of 90%, for every additional unit in the world happiness index score, the effect size of a study rose by 0.05 (90% CI = −0.01, 0.11). For life expectancy, a positive correlation was shown between life expectancy and self-reported health effect sizes: for every year increase in life expectancy across countries, the association between hobby engagement and self-reported health was 0.01 points larger (coefficient = 0.01; 95% CI = 0.01, 0.02). No associations were found for other outcomes (Extended Data Fig. 7).

Finally, we explored whether these country-level factors could moderate the relationship between hobby engagement and mental wellbeing. When interacting hobby engagement with country-level factors in multilevel models, there was a small moderating effect of Gini index, the world happiness index, country wealth and life expectancy on the associations between hobbies and depression, life satisfaction and self-reported health, but not on happiness (Extended Data Fig. 2).

### Sensitivity analyses

When using multiple imputation to account for missing data, results were largely replicated (Supplementary Table 2). When including respondents aged 55+ (except for Japan where all participants were aged 65+), the evidence for longitudinal associations between hobby engagement and the outcomes across countries became stronger, likely because of the increase in the number of respondents (Supplementary Table 3).

**Extended Data Fig. 8:**
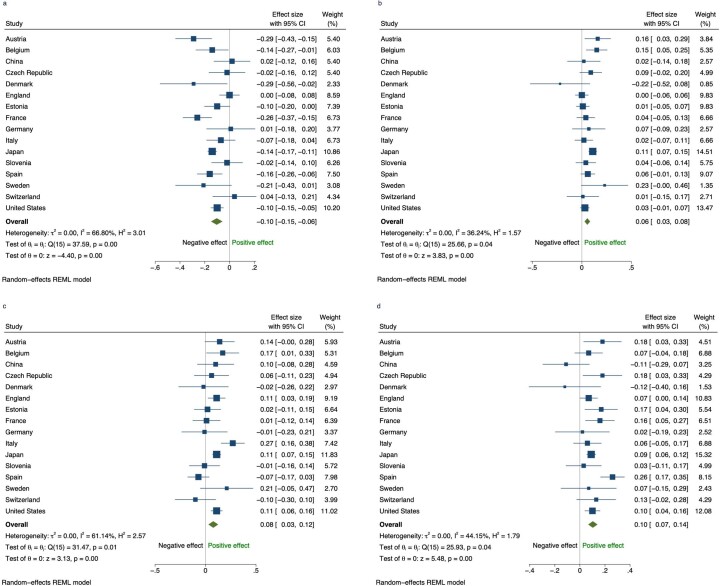
Fixed effects analyses of hobbies and mental wellbeing outcomes for females. N study = 16. Data were first analysed separately for each country using fixed effects regression. All models controlled for all time-constant variables and time-varying variables including age, partnership status, number of people living in the household, employment status, household income, housing tenure, long-standing mental/physical conditions, difficulties with daily activities (ADLs), and difficulties with instrumental activities of daily living (IADLs). The findings were then pooled into multi-national meta-analyses using the random effect model to estimate the overall effect sizes for all outcomes. Between-study heterogeneity was estimated using the algorithm of the restricted maximum likelihood and was assessed using I^2^ and H^2^ statistics. I^2^ indicates the percentage of variability in the effect size that is caused by between-study heterogeneity, rather than by sampling error. H^2^ statistics describes the ratio of the observed variation and the expected variance due to sampling error. Given that some of the analyses had more participants than others and thus had lower sampling variability and more precise estimates, the meta-analysis was weighted. Studies with a greater number of respondents were given more weight than studies with a small number of respondents. These were relative weights that summed to 100. Data are presented as fixed effects coefficients and 95% confidence intervals. The overall effect size and its width should have accounted for the between-study variance, the number of studies, the precision of the study-specific estimates (or ‘effect sizes’) and the significance level. **a**. Depressive symptoms. **b**. Self-reported health. **c**. Happiness. **d**. Life satisfaction.

**Extended Data Fig. 9:**
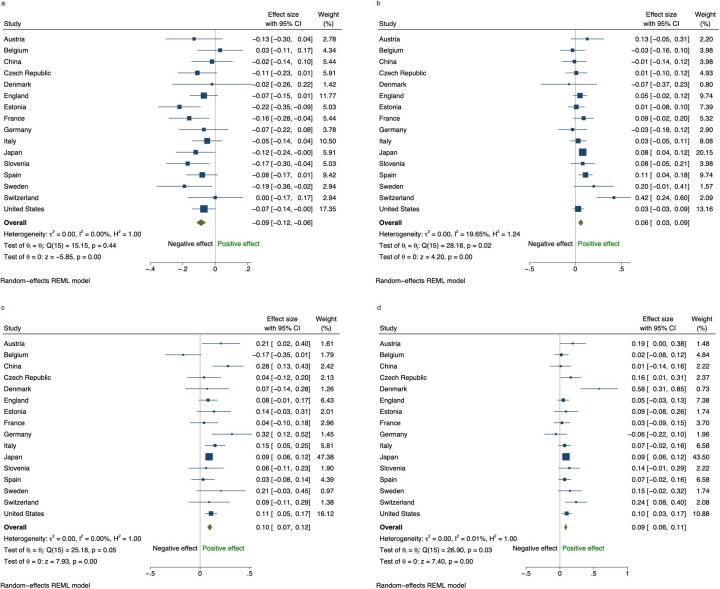
Fixed effects analyses of hobbies and mental wellbeing outcomes for males. N study = 16. Data were first analysed separately for each country using fixed effects regression. All models controlled for all time-constant variables and time-varying variables including age, partnership status, number of people living in the household, employment status, household income, housing tenure, long-standing mental/physical conditions, difficulties with daily activities (ADLs), and difficulties with instrumental activities of daily living (IADLs). The findings were then pooled into multi-national meta-analyses using the random effect model to estimate the overall effect sizes for all outcomes. Between-study heterogeneity was estimated using the algorithm of the restricted maximum likelihood and was assessed using I^2^ and H^2^ statistics. I^2^ indicates the percentage of variability in the effect size that is caused by between-study heterogeneity, rather than by sampling error. H^2^ statistics describes the ratio of the observed variation and the expected variance due to sampling error. Given that some of the analyses had more participants than others and thus had lower sampling variability and more precise estimates, the meta-analysis was weighted. Studies with a greater number of respondents were given more weight than studies with a small number of respondents. These were relative weights that summed to 100. Data are presented as fixed effects coefficients and 95% confidence intervals. The overall effect size and its width should have accounted for the between-study variance, the number of studies, the precision of the study-specific estimates (or ‘effect sizes’) and the significance level. **a**. Depressive symptoms. **b**. Self-reported health. **c**. Happiness. **d**. Life satisfaction.

When analyses were stratified by gender, we found some variations between countries. However, pooled effect sizes from meta-analysis showed that engagement in hobbies remained beneficial for both females (depressive symptoms: pooled coefficient = −0.10; 95% CI = −0.15, −0.06; self-reported health: pooled coefficient = 0.06; 95% CI = 0.03, 0.08; happiness: pooled coefficient = 0.08; 95% CI = 0.03, 0.12; life satisfaction: pooled coefficient = 0.10; 95% CI = 0.07, 0.14) and males (depressive symptoms: pooled coefficient = −0.09; 95% CI = −0.12, −0.06; self-reported health: pooled coefficient = 0.06; 95% CI = 0.03, 0.09; happiness: pooled coefficient = 0.10; 95% CI = 0.07, 0.12; life satisfaction: pooled coefficient = 0.09; 95% CI = 0.06, 0.11) (Extended Data Fig. 8 for female and Extended Data Fig. 9 for male).

**Extended Data Fig. 10:**
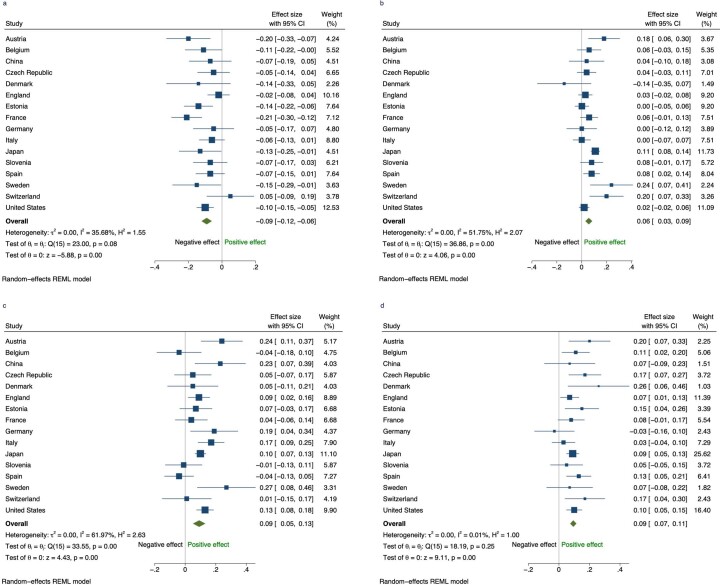
Fixed effects analyses of hobbies and mental wellbeing outcomes for adults who were retired. N study = 16. Data were first analysed separately for each country using fixed effects regression. All models controlled for all time-constant variables and time-varying variables including age, partnership status, number of people living in the household, employment status, household income, housing tenure, long-standing mental/physical conditions, difficulties with daily activities (ADLs), and difficulties with instrumental activities of daily living (IADLs). The findings were then pooled into multi-national meta-analyses using the random effect model to estimate the overall effect sizes for all outcomes. Between-study heterogeneity was estimated using the algorithm of the restricted maximum likelihood and was assessed using I^2^ and H^2^ statistics. I^2^ indicates the percentage of variability in the effect size that is caused by between-study heterogeneity, rather than by sampling error. H^2^ statistics describes the ratio of the observed variation and the expected variance due to sampling error. Given that some of the analyses had more participants than others and thus had lower sampling variability and more precise estimates, the meta-analysis was weighted. Studies with a greater number of respondents were given more weight than studies with a small number of respondents. These were relative weights that summed to 100. Data are presented as fixed effects coefficients and 95% confidence intervals. The overall effect size and its width should have accounted for the between-study variance, the number of studies, the precision of the study-specific estimates (or ‘effect sizes’) and the significance level. **a**. Depressive symptoms. **b**. Self-reported health. **c**. Happiness. **d**. Life satisfaction.

The potential positive effects of hobby engagement remained when only considering respondents who were retired (depressive symptoms: pooled coefficient = −0.09; 95% CI = −0.12, −0.06; self-reported health: pooled coefficient = 0.06; 95% CI = 0.03, 0.09; happiness: pooled coefficient = 0.09; 95% CI = 0.05, 0.13; life satisfaction: pooled coefficient = 0.09; 95% CI = 0.07, 0.11) (Extended Data Fig. 10). Further, multilevel model analyses also showed no moderating effects of national pension age (Extended Data Fig. 2f).

To assess whether the type of hobbies measured (binary measure or index created from a list of options) was responsible for differences in effect sizes between studies, a subgroup meta-analysis was conducted. Pooled analyses showed no subgroup differences between the measures; hobbies continued to associate with all outcomes (*P* > 0.05; Supplementary Table 4). We also included a variable capturing the type of hobby measure within the multilevel models of the merged datasets, but results were unaffected, suggesting measurement bias did not underlie the findings (Extended Data Fig. 2). Even when excluding CHARLS data (which focused exclusively on social hobbies rather than solitary ones) in our meta-analyses of analyses exploring directionality, the results were consistent (Extended Data Fig. 1).

## Discussion

This study compared longitudinal associations between hobby engagement and multidimensional aspects of mental wellbeing across 16 countries. The prevalence of hobby engagement varied substantially across countries, from countries where only one in two people had a hobby (for example, 51.0% of the Spanish respondents) to countries where hobby engagement was ubiquitous (for example, 96.0% of the Danish respondents). Meta-analysis of the findings revealed that having a hobby was associated with fewer depressive symptoms, better self-reported health, more happiness and higher life satisfaction, with life satisfaction most consistently related to hobbies. Looking at the direction of these associations, increased hobby engagement predicted subsequent decreases in depressive symptoms and increased self-reported health, happiness and life satisfaction. There was little variance in findings among countries, suggesting a relative universality of response. However, on average, more adults aged 65+ had hobbies in countries with higher world happiness index score and life expectancy, and the relationship between hobby engagement and life satisfaction and self-reported health was slightly stronger in such countries. Sensitivity analysis showed that findings did not vary by gender or retirement status, nor by country-level retirement age.

Our findings are in line with various cross-disciplinary international literature indicating that having a hobby may enhance mental wellbeing among adults aged 65+, but they present an advance on past literature in several ways. First, the results provide evidence for the consistency in such findings across cultural settings and countries, highlighting the relevance to global public health policies and practices. Of the four outcomes, hobby engagement has the most consistent association with life satisfaction; a subjective evaluation of one’s social, emotional and physical wellbeing that can be independent of ‘objective’ health status or functional ability, which tend to decline with age^31^. Hobbies could contribute to older adults’ life satisfaction through many mechanisms, including feeling in control of their minds and bodies, finding a purpose in life and feeling competent in tackling daily issues^26^. Our temporal analyses showed that these associations were not merely the result of good psychological health predicting hobby engagement. In actuality, the relationship between hobbies and mental wellbeing is likely bidirectional, because theoretical work applying lenses from complex adaptive systems science to leisure engagement and health has posited constant positive and negative feedback loops between leisure behaviors and health outcomes^26^. But our directionality findings are encouraging because they suggest that experimental efforts to increase hobby engagement may have the potential to alter subsequent mental wellbeing. Indeed, the association of hobbies with life satisfaction is particularly promising given that it was seen not only in healthier respondents, but also in respondents such as those in the USA where a very high proportion of the respondents were living with long-standing mental or physical health conditions and where psychosocial interventions could be even more relevant.

However, it is also relevant to consider why, even in the context of notable meta-analytic findings, associations between hobby engagement and mental wellbeing showed some variation across countries. The results from meta-regression analyses showed that there may be a positive correlation between world happiness index score and effect sizes for life satisfaction, suggesting that the effect sizes of studies increase according to how happy people are on a country level. Similarly, the effect sizes for self-reported health were also generally larger for countries with higher life expectancy. Individuals living in countries with higher life expectancies or happiness levels may be more likely to have a hobby (for example, Denmark, Sweden and Switzerland; Fig. 3c,d), which may have inflated the size of the coefficients. However, there are some exceptions. For example, although people living in Spain had a lower engagement rate comparatively, the strength of the association between hobby engagement and life satisfaction was similar to that for countries with much higher engagement rates (including Austria, Czech Republic and Switzerland). This suggests the health benefits of hobbies (at least for life satisfaction) are not simply driven by the high prevalence of engagement rates, but can also be found in countries where hobbies are less popular. Similarly, the longitudinal associations between hobbies and the outcomes continued to be found in countries with lower world happiness index scores (for example, Japan and China) as well as countries with lower life expectancy (for example, the USA). In countries with higher happiness levels and life expectancy, there may be fewer psychological barriers to hobby engagement in the first place and stronger positive feedback loops supporting the translation of this engagement into causal mechanisms that both support mental wellbeing and additionally contribute to the maintenance of and even increase in the original salutogenic hobby behavior. However, less than 9% of the variance in findings was explained by country. So, taken together, our results suggest that having a hobby may have the potential to be associated with improvements in health among the older population cross-culturally. This supports theories from anthropology, evolutionary psychology and sociology that have focused on the potential adaptive benefits of hobby engagement, seeing pleasure as a by-product but other functions such as developing attention and cognition, social bonding and societal cohesion, communication and knowledge, and adaptation as important to species survival^32–34^.

The differential participation rates in hobbies across countries must be cautiously interpreted given that questions about hobbies varied in style and in length of time asked about within the datasets. In particular, China’s lower hobby rate may be partly influenced by the questions focusing largely on social hobbies (hence our additional sensitivity analyses excluding China’s data from meta-analysis). Nonetheless, even among countries with identical questions on hobbies (such as the 12 countries within the SHARE dataset), there was substantial variation in participation rates. This may be a result of greater barriers to engagement in some countries. Indeed, hobbies are often perceived as an ‘asset’ possessed by older people who are healthier, happier and wealthier. Within countries, previous literature has highlighted a social gradient in hobby engagement, where gender, social class, ethnicity and health conditions could influence the likelihood of engagement among adults aged 50+ (ref. ^35^). Between-country comparisons found greater hobby engagement rates in more affluent countries. Differences in hobby participation are thus concerning, because they could contribute to or exacerbate health inequalities both within and between countries. As a result, in working to capitalize on the findings presented here, a systematic approach should be taken, considering both how to address individual-level barriers to hobby engagement that adults aged 65+ may face, as well as considering how societal interventions could be designed to build stronger relationships at a public health level between hobby engagement and mental wellbeing outcomes. Public health strategies such as social prescribing schemes including in the UK, USA, Japan and parts of Europe have focused on building hobby engagement into healthcare services, providing new referral pathways that can help to address existing individual and societal barriers to engagement, positively influencing motivations and propensity to engage among older populations, and in turn providing opportunities to strengthen the associations between hobby and health outcomes.

Our findings have policy and health implications for adults aged 65+, especially those who are retired (between 56.2% and 98.8% of our respondents). Contemporary life-course research has demonstrated that the concept of aging has shifted from seniority to an emphasis on lifestyle and consumption including expenditures for services and healthy goods^36^. This aligns with the idea of ‘the third age’ emphasized in previous research, which suggests that older adults who enter the retirement age are now presented opportunities for self-development and are liberated from the previous label of an ‘old age pensioner’ and from ‘the fourth age’ of decline and dependency^36^. As suggested in our findings, hobbies such as physical activity, arts and cultural engagement, and social and community participation have the potential to lengthen ‘the third age’ period and make it one of ‘productive aging’ through protecting against age-related declines in mental health and enhancing wellbeing, which have profound consequences for morbidity and mortality.

There are many strengths in this study including the use of five national longitudinal studies containing data from 16 nations. The study also uses population surveys to compare hobby engagement rates internationally, as well as assessing the strengths of the associations against population statistics relating to country wealth, Gini index, world happiness index score and life expectancy. In addition, fixed effects analyses allowed us to explore how changes in hobbies were associated with changes in the mental wellbeing outcomes, while adjusting for all time-constant variables (regardless of whether they were observed) and important time-varying variables.

However, the study is not without limitations. Because of the use of observational data, causality cannot be established even with sophisticated longitudinal data analysis modeling. Further, although there was overall relative homogeneity in the way questions about hobbies were asked and sub-questions were collapsed into a binary indicator, some countries chose to list hobby examples, whereas others did not, which may have led to differences in interpretation of the question by the respondents. However, no differences in associations between hobbies and outcomes were found with different measures (as shown in Supplementary Table 4) and the use of only 5 studies for 16 countries limited the amount of heterogeneity in the measures. Relatedly, the reference period measuring hobby engagement rate varied across the longitudinal datasets, although we still found some engagement variations between countries with the same reference period measure.

Future research is needed to consider the types, frequency and length of hobby engagement in different countries, as well as whether modulation of specific types of hobby engagement (such as the presence or absence of physical activity or social interaction) differentially affect outcomes^25^. It will also be necessary to examine further whether key benefits of hobby engagement are derived from the activities themselves or additionally from time spent on hobbies displacing time that otherwise could be spent on less salutogenic activities including chores, work or procrastination. In addition, our analysis did not explore other intraindividual factors that are largely time-constant but may have some limited variability. Future studies may wish to use datasets with more interview waves that might capture this variability over time to explore the role of such factors as moderators of effects. Finally, natural experiments such as changes in leisure or retirement policies or behaviors (for example, as the result of major financial upheavals within countries) are encouraged to explore potential causal effects of hobbies on mental wellbeing in more detail.

The cross-national mental wellbeing benefits of hobby engagement reported here suggest that facilitating greater opportunities for engagement across demographic groups and between countries should be a priority in efforts to increase healthy life expectancy and relieve the increased burden of aging populations on healthcare systems internationally. Results from this study could also be used as evidence when formulating and developing schemes to increase equity of access to leisure activities among older adults across demographic groups and between countries, as well as in integrating psychosocial interventions into health services or public health strategies (for example, through social prescribing schemes) to reduce morbidity, mortality and healthcare burden, and enhance aging experiences among older adults.

## Methods

### Data

#### ELSA

ELSA started in 2002–2023 and follows over 11,000 participants aged 50+ living in England every 2 years^37^. In this study, to be in line with the other datasets, we extracted a pool of respondents aged 65+ who responded in Waves 7 (2014–2015; response rate = 78.3%–81.4%), 8 (2016–2017; response rate = 82.4%) and 9 (2018–2019; response rate = 79.5%) where hobby engagement and outcome variables were measured. We considered only respondents who provided data across all measures. This resulted in 10,876 observations from 4,267 participants (2.5 per person, ranging from 2 to 3).

#### JAGES

JAGES is a large-scale population-based longitudinal study about aging established in 2010, mainly collected through self-administered mail surveys, targeting older people aged 65+ who do not receive long-term care insurance benefits^38^. JAGES has conducted a joint survey with municipalities that are the public insurers of long-term care insurance every 3–4 years: Wave 1 (2010–2011) to Wave 4 (2019–2020). This study used data from Waves 2, 3 and 4 (30–64 municipalities; response rate = 52.4%–71.1%). Of the respondents, those with complete data on hobby engagement and health outcomes in at least two waves were considered. This resulted in 125,901 observations from 57,051 participants (2.4 person, ranging from 2 to 3).

#### HRS

HRS is a national cohort study of more than 37,000 individuals over the age of 50 in the USA^39^. The study was initiated by the National Institute on Aging and conducted by the Institute for Social Research at the University of Michigan to track the baby boom generation’s transition from work to retirement. The initial HRS cohort was interviewed for the first time in 1992 and followed up every 2 years, with other studies and younger cohorts merged with the initial pool of respondents. Together, these studies create a group of fully representative respondents aged over 50 in the USA. Further details on study design are reported elsewhere^39^. We used data from HRS Waves 9–14 at which participation in a hobby was measured (2008–2018). At each wave, a rotating random 50% subgroup of respondents was invited to an enhanced interview and given a Leave Behind Psychosocial and Lifestyle Questionnaire to complete and return by mail, which included questions on participation in community arts groups and mental wellbeing^40^. Participants were eligible to complete this psychosocial questionnaire every 4 years. Response rates in each year varied from 62% to 85%. We restricted the respondents to those aged 65+, with complete data on hobby engagement and mental wellbeing outcomes in at least two waves and no missing data on time-varying covariates. This resulted in 14,989 observations from 6,204 participants (2.4 observations per person, range 2–3).

#### SHARE

SHARE is the largest pan-European social science panel study providing internationally comparable longitudinal micro data on the population aged 50+ and currently includes eight waves with data collection starting in 2004. SHARE contains both the participation of respondents in their baseline and refreshment interview to account for a reduction in the number of respondents due to panel attrition. SHARE has original core questionnaires as well as retrospective questionnaires (SHARELIFE, in Waves 3 and 8). In Waves 3 and 8, respondents answering the retrospective questionnaire were asked to answer a reduced core questionnaire with less information, justifying the use of Waves 4, 5 and 6 in this study. Data information for these three waves is available for twelve countries. Data were not available over these three waves for Croatia, Greece, Hungary, Israel, Luxembourg, the Netherlands, Poland and Portugal. The analytical pool of respondents by country, including nonresponse at baseline, is: 2,524 in Austria; 2,304 in Belgium; 2,664 in Czech Republic; 1,006 in Denmark; 3,584 in Estonia; 2,705 in France; 966 in Germany; 1,915 in Italy; 1,272 in Slovenia; 2,099 in Spain; 1,315 in Sweden; and 1,776 in Switzerland.

#### CHARLS

CHARLS is a national cohort study of Chinese residents aged 45+ (ref. ^41^). The baseline survey started in 2011 and has been followed up every 2 years (in 2013 and 2015). Multistage probability sampling was used for a selection of respondents. The baseline included 17,708 individuals, and the response rates were over 80% in all three waves (Wave 1 = 80.5%, Wave 2 = 82.6% and Wave 3 = 82.1%). The study considered only participants who responded to all measures, resulting in 3,440 observations from 1,611 participants (2.1 observations per person, range 2–3).

### Measures

#### Hobby and mental wellbeing

Our measures of hobby and mental wellbeing are shown in Supplementary Table 5, which presents the exact question-wording and item responses across datasets. Hobbies and mental wellbeing outcomes were time-varying variables. The analysis will focus on four types of mental wellbeing: depressive symptoms, self-reported health, happiness and life satisfaction. The measure items and response categories vary somewhat by country, reflecting cultural differences across the 16 nations. Therefore, to ensure the data were comparable, we harmonized and recoded all variables, and standardized the outcome variables. We created a binary indicator of hobby engagement (yes, no) in each country. Nonetheless, care needs to be taken in comparing the proportion of hobby engaged and levels of various mental wellbeing outcomes across countries.

#### Time-varying covariates

Nine time-varying variables that might confound observed associations between hobby and mental wellbeing were identified for the analysis. These included demographic characteristics: age (a continuous variable), partnership status (living with a partner/spouse versus not living with a partner/spouse), number of people living in the household (a continuous variable); socioeconomic position: employment status (working versus not working), household income (a continuous variable), housing tenure (homeowner versus not a homeowner); and health profiles: long-standing mental/physical conditions (yes versus no), difficulties with daily activities (ADL) (with difficulties versus without difficulties) and difficulties with instrumental activities of daily living (IADL; a continuous variable).

### Statistical analysis

In the first instance, data were analyzed separately for each country using fixed effects regression. Fixed effects regression is a longitudinal data method that tests within-individual variation, meaning that each individual is compared with themselves over time. Such a model automatically controls for all time-invariant variables such as age, gender, genetics, personality, socioeconomic status, education, area of dwelling, past life experiences, past mental health and medical history, even if they are unobserved, as well as controlling for identified time-varying covariates. For this reason, fixed effects regression is considered to be more robust than traditional regression models in exploring how changes in the predictor are associated with the changes in the outcomes.

We pooled our findings into multinational meta-analyses using the random effects model to estimate the overall effect sizes for all outcomes. Pooled effect sizes and 95% CI were reported. Between-study heterogeneity was estimated using the algorithm of the restricted maximum likelihood and was assessed using *I*^2^ and *H*^2^ statistics. *I*^2^ indicates the percentage of variability in the effect size that is caused by between-study heterogeneity, rather than by sampling error^42^. A value of *I*^2 ^> 50% indicates heterogeneity^42^. Similarly, the *H*^2^ statistic describes the ratio of the observed variation and the expected variance caused by sampling error^42^. A value of *H*^2 ^> 1 indicates the presence of between-study heterogeneity^42^. Given that some of the analyses had more participants than others and thus had lower sampling variability and more precise estimates, the meta-analysis was weighted. Studies with a greater number of respondents were given more weight than studies with a small number of respondents. These were relative weights that summed to 100. The overall effect size and its width should have accounted for the between-study variance, the number of studies, the precision of the study-specific estimates (or ‘effect sizes’) and the significance level. We also conducted a subgroup analysis by hobbies measures to explore whether the differences in effect sizes might have been attributed to the way in which questions on hobbies were asked in each longitudinal study (a binary measure versus index created from a list options). We further ran meta-regressions to explore the heterogeneity variance in our meta-analysis using five country-level factors: the prevalence of hobby engagement, country wealth measured by gross domestic product per capita^28^, world happiness index score^27^, life expectancy^29^ and the Gini index measuring income inequality within a nation^30^.

Although fixed effects analyses explored the longitudinal associations between changes in hobby engagement and changes in mental wellbeing outcomes, they cannot test the direction of these relationships. We therefore performed two further sets of analyses. First, we ran OLS regressions estimating the association between hobbies measured at Time 1 and outcomes measured at Time 2, while controlling for baseline outcomes and covariates. The covariates included age, gender, the number of people living in the household, partnership status, household income, housing tenure, employment status, educational level, long-term mental/physical conditions, ADLs and IADLs. Results from these analyses were then pooled into meta-analysis as described above. Respondents from China were dropped because of the inclusion of only social hobbies. Second, we tested the consistency of these findings using a different statistical approach—lagged fixed effects models using an Arellano–Bond estimator model—on the ELSA dataset where there were sufficient repeated waves (nine available). The Arellano–Bond estimator is considered an extension of the fixed effects model, which uses a first-difference model and includes lags of the outcome variable as instruments for the first difference^43^. This model takes account of previous changes in mental wellbeing outcomes over time to estimate the effect of hobby on subsequent changes in the outcomes, while accounting for differences in individual characteristics. However, the model requires multiple waves of data and consistency in measures across every wave. Not all of the datasets in our analyses met these requirements, so we performed the Arellano–Bond estimator analyses solely on ELSA, which is one of the earliest aging longitudinal studies with a longer follow-up period and more consistent measures than many of the other datasets. By applying the Arellano–Bond estimator to this dataset, we were able to ascertain whether the findings matched those from the OLS regressions and confirm that any findings from the OLS regressions were not merely the result of a less sophisticated statistical approach. In the analysis, all models were fully adjusted without any age restriction to allow sufficient statistical power for use of the Arellano–Bond estimator.

For both fixed effects and regression analyses, listwise deletion was applied to handle missing data. The proportion of missingness was as follows: Austria (20.6%), Belgium (39.6%), China (69.7%; mainly because of missingness in household wealth, hobby and life satisfaction), Czech Republic (34.8%), Denmark (56.6%), England (15.1%), Estonia (20.8%), France (21.2%), Germany (69.1%), Italy (51.3%), Japan (54.0%), Slovenia (54.8%), Spain (53.8%), Sweden (62.5%), Switzerland (19.3%) and the USA (15.7%). In our main analysis, we present coefficients and 95% CIs to show the relationship between hobby engagement and the outcomes across countries after adjustment for time-varying covariates. Stata v.17 was used for the analyses.

To explore whether country-level factors could moderate the relationship between hobby engagement and mental wellbeing, we pooled data from four longitudinal datasets and undertook multilevel analyses (JAGES was not available because of data restriction). The models were adjusted for interview waves, type of hobby measure, age, gender, the number of people living in the household, partnership status, household income, housing tenure, employment status, educational level, long-term mental/physical conditions, ADLs and IADLs.

Finally, we performed a set of sensitivity analyses to explore the robustness of the associations between hobby engagement and mental wellbeing:

1. To check that missing data did not influence our findings, we re-ran the analysis after using multiple imputation by chained equations to impute missing data on hobby engagement, mental wellbeing outcomes and time-varying covariates across all included waves.
2. The main analysis considered only respondents aged 65+ to allow for comparison across all datasets, but this significantly restricted the number of respondents in the ELSA, HRS, SHARE and CHARLS data. This might reduce statistical power. To check the robustness of our main results, we replicated the analysis using these four datasets and extended the pool of respondents to those who were aged 55+.
3. To test for the consistency of the association between hobby engagement and mental wellbeing across different population groups, we stratified our respondents by gender (female and male) and restricted our respondents to those who were retired.

### Ethics and inclusion statement

This research analyzed five large and longitudinal datasets across England, the USA, Europe, Japan and China, and collaborated with local researchers throughout the research process to ensure its local relevance. H.W.M. and D.F. are from the UK, J.K.B. is also from the UK but her work has largely focused on the US context; J.W. is based in Belgium; T.N., K.K. and T.S. are from Japan; and Q.G. is based in the UK and originally from China. Roles and responsibilities were agreed among authors ahead of the research.

This research is locally relevant to all studied countries given that it shows individual findings by country, while aggregating them to provide more conclusive evidence on the psychological benefits of hobby engagement for older adults. These findings can provide local decision-makers with data that could support the drafting of recommendations on supporting healthy aging though encouraging hobby uptake. The research result does not result in stigmatization, incrimination, discrimination or otherwise personal risk to participants. The research did not involve any health, safety, security or other risk to researchers. No biological materials, cultural artifacts or associated traditional knowledge were transferred out of any country. The authors have undertaken research relevant to the study.

### Ethics approval

#### ELSA

ELSA Wave 9 received ethical approval from the South Central—Berkshire Research Ethics Committee on 10 May 2018 (17/SC/0588). ELSA Wave 8 received ethical approval from the South Central—Berkshire Research Ethics Committee on 23 September 2015 (15/SC/0526). ELSA Wave 7 received ethical approval from the National Research Ethics Service (NRES) Committee South Central—Berkshire on 28 November 2013 (13/SC/0532). ELSA Wave 6 received ethical approval from the NRES Committee South Central—Berkshire on 28 November 2012 (11/SC/0374). ELSA Wave 5 received ethical approval from the Berkshire Research Ethics Committee on 21 December 2009 (09/H0505/124). ELSA Wave 4 received ethical approval from the National Hospital for Neurology and Neurosurgery and Institute of Neurology Joint Research Ethics Committee on 12 October 2007 (07/H0716/48). ELSA Wave 3 received ethical approval from the London Multi-Centre Research Ethics Committee on 27 October 2005 (05/MRE02/63). ELSA Wave 2 received ethical approval from the London Multi-Centre Research Ethics Committee on 12 August 2004 (MREC/04/2/006). ELSA Wave 1 received ethical approval from the London Multi-Centre Research Ethics Committee on 7 February 2002 (MREC/01/2/91). All participants provided informed written consent.

#### JAGES

JAGES received ethical approval from Nihon Fukushi University (no. 10-05), Chiba University (no. 2493) and the National Center for Geriatrics and Gerontology (no. 992), and all participants provided informed written consent.

#### HRS

Ethical approval for HRS was obtained from the University of Michigan Institutional Review Board. All participants gave informed written consent.

#### SHARE

SHARE received ethical approval from the Ethics Council of the Max Planck Society and all participants provided informed written consent.

#### CHARLS

CHARLS received ethical approval from the Biomedical Ethics Review Committee of Peking University (IRB00001052-11015) and all participants provided informed written consent.

### Reporting summary

Further information on research design is available in the Nature Portfolio Reporting Summary linked to this article.

## Online content

Any methods, additional references, Nature Portfolio reporting summaries, source data, extended data, supplementary information, acknowledgements, peer review information; details of author contributions and competing interests; and statements of data and code availability are available at 10.1038/s41591-023-02506-1.

## Supplementary information

Supplementary Information Supplementary Tables 1–5.

Reporting Summary

## Data Availability

The English Longitudinal Study of Ageing (ELSA) can be accessed via the UK Data Service: https://beta.ukdataservice.ac.uk/datacatalogue/series/series?id=200011. The Health and Retirement Study (HRS) can be accessed via the RAND Center for the Study of Aging: https://hrsdata.isr.umich.edu/data-products/rand. The Survey of Health, Ageing and Retirement in Europe (SHARE) can be accessed via the SHARE Research Data Center: http://www.share-project.org/data-access.html. The China Health and Retirement Longitudinal Study (CHARLS) can be accessed via the National School of Development, Peking University: https://charls.charlsdata.com/pages/data/111/en.html. Restrictions to access data of Japan Gerontological Evaluation Study (JAGES) applied. For researchers who wish to use the data, please contact the JAGES Data Administration Office at dataadmin.ml@jages.net. Non-JAGES research members may be required to include JAGES members in their project or co-authors in research papers depending on the study topic or data used.

## Code availability

All code used for these analyses is publicly available online: https://osf.io/84xzu/.
