# Supplementary material for: Hobby engagement and mental wellbeing among people aged 65 years and older in 16 countries
Source: Nat Med. 2023 Sep 11;29(9):2233–40. doi: 10.1038/s41591-023-02506-1 (PMC10504079; doi:10.1038/s41591-023-02506-1)
Supplement: Supplementary file 1 — Supplementary Tables 1–5. [file 41591_2023_2506_MOESM1_ESM.pdf]

nature medicine

Article https://doi.org/10.1038/s41591-023-02506-1

Hobby engagement and mental wellbeing among people aged 65 years and older in 16 countries

In the format provided by the
authors and unedited

Supplementary Information

Supplementary Table 1 | Directionality testing using the Arellano-Bond estimator model

	Coef	95%CI	p-value	Hansen test	Difference-Hansen	N obs	N groups
Depressive symptoms	**-0.38**	**-0.63, -0.12**	**0.003**	95.13 (73)	37.5 (23)	31,615	9,258
Self-reported health	**0.73**	**0.47, 0.99**	**<0.001**	63.67 (41)	8.01 (11)	25,664	9,100
Happiness[1]	**0.36**	**0.01, 0.71**	**0.043**	40.44 (22)	12.48 (7)	24,506	8,922
Life satisfaction	0.19	-0.03, 0.41	0.098	95.30 (73)	47.42 (23)	27,345	8,189

Note: Data from the English Longitudinal Study of Ageing Waves 1-9 were used. In this analysis, the Arellano-Bond estimator model was applied to test whether hobbies predicted subsequent mental health outcomes. No age restriction was applied to allow sufficient statistical power to detect the directional relationships between hobby engagement and mental health outcomes. The respondents were aged 50 and above. [1]The question on happiness was asked from Wave 5 so the model only used data from Waves 5-9. The number of observations/groups varied across outcomes due to different lagged variables. The models controlled for all time-constant variables and time-varying variables including age, partnership status, number of people living in the household, employment status, household income, housing tenure, long-standing mental/physical conditions, difficulties with daily activities (ADLs), and difficulties with instrumental activities of daily living (IADLs). Bold values denote statistical significance at the p<0.05 level.

Supplementary Table 2 | Fixed effects analyses with multiple imputations

	Depressive symptoms	Self-reported health	Happiness	Life satisfaction
	Coef (95%CI)	Coef (95%CI)	Coef (95%CI)	Coef (95%CI)
Austria (N=3,178)	**-0.27**	**0.11**	0.11	**0.18**
	**(-0.40, -0.14)**	**(0.01, 0.21)**	(-0.01, 0.25)	**(0.05, 0.30)**
Belgium (N=3,814)	**-0.11**	**0.09**	0.03	0.04
	**(-0.21, -0.01)**	**(0.01, 0.18)**	(-0.09, 0.15)	(-0.04, 0.12)
China (N=5,316)	**-0.20**	-0.01	**0.29**	0.01
	**(-0.30,-0.10)**	(-0.08,0.06)	**(0.20,0.39)**	(-0.11,0.13)
Czech Republic (N=4,086)	-0.04	0.04	0.06	**0.19**
	(-0.16, 0.80)	(-0.03, 0.12)	(-0.07, 0.19)	**(0.07, 0.31)**
Denmark (N=2,317)	**-0.22**	-0.07	0.09	**0.41**
	**(-0.49, 0.05)**	(-0.28, 0.13)	(-0.07, 0.24)	**(0.22, 0.61)**
England (N=5,026)	-0.02	0.02	**0.08**	**0.06**
	(-0.08, 0.03)	(-0.02, 0.07)	**(0.02, 0.14)**	**(0.01, 0.12)**
Estonia (N=4,528)	**-0.17**	0.01	0.05	**0.17**
	**(-0.26, -0.08)**	(-0.05, 0.06)	(-0.05, 0.14)	**(0.06, 0.28)**
France (N=3,431)	**-0.21**	0.01	0.02	**0.09**
	**(-0.32, -0.10)**	(-0.49, 0.60)	(-0.08, 0.12)	**(0.01, 0.17)**
Germany (N=3,128)	-0.05	0.07	0.13	-0.02
	(-0.17, 0.07)	(-0.05, 0.18)	(-0.02, 0.28)	(-0.17, 0.14)
Italy (N=3,932)	**-0.11**	0.05	**0.23**	**0.09**
	**(-0.19, 0.03)**	(-0.01, 0.11)	**(0.15, 0.31)**	**(0.01, 0.18)**
Japan (N=124,057)	**-0.13**	**0.10**	**0.09**	**0.09**
	**(-0.15, -0.11)**	**(0.08, 0.12)**	**(0.07, 0.10)**	**(0.07, 0.11)**
Slovenia (N=2,816)	**-0.11**	**0.10**	0.08	0.11
	**(-0.21, -0.02)**	**(0.02, 0.18)**	(-0.03, 0.19)	(-0.01, 0.22)
Spain (N=4,547)	**-0.11**	**0.12**	**-0.12**	**0.13**
	**(-0.18, -0.03)**	**(0.07, 0.17)**	**(-0.20, -0.04)**	**(0.05, 0.20)**
Sweden (N=3,508)	**-0.12**	**0.16**	**0.24**	0.10
	**(-0.26, 0.03)**	**(0.01, 0.31)**	**(0.07, 0.42)**	(-0.05, 0.24)
Switzerland (N=2,201)	0.02	**0.20**	-0.02	**0.19**
	(-0.10, 0.15)	**(0.08, 0.32)**	(-0.16, 0.13)	**(0.08, 0.31)**
United States (N=7,362)	**-0.07**	0.03	**0.09**	**0.10**
	**(-0.13, -0.02)**	(-0.01, 0.07)	**(0.04, 0.14)**	**(0.03, 0.16)**

Note: The models controlled for all time-constant variables and time-varying variables including age, partnership status, number of people living in the household, employment status, household income, housing tenure, long-standing mental/physical conditions, difficulties with daily activities (ADLs), and difficulties with instrumental activities of daily living (IADLs). Bold values denote statistical significance at the p<0.05 level.

Supplementary Table 3 | Fixed effects analyses for older adults aged 55 and above

	Depressive symptoms	Self-reported health	Happiness	Life satisfaction
	Coef (95%CI)	Coef (95%CI)	Coef (95%CI)	Coef (95%CI)
Austria (N=4,838)	**-0.15**	**0.13**	**0.14**	**0.17**
	**(-0.23, -0.06)**	**(0.05, 0.21)**	**(0.05, 0.23)**	**(0.08, 0.26)**
Belgium (N=6,428)	**-0.08**	**0.07**	0.07	**0.06**
	**(-0.15, -0.001)**	**(0.004, 0.13)**	(-0.02, 0.16)	**(0.004, 0.12)**
China (N= 4,875)	-0.03	0.02	**0.22**	0.03
	(-0.09,0.02)	(-0.04,0.09)	**(0.15,0.30)**	(-0.04,0.10)
Czech Republic (N=6,412)	**-0.10**	0.03	0.06	**0.09**
	**(-0.17, -0.03)**	(-0.03, 0.09)	(-0.03, 0.14)	**(0.01, 0.17)**
Denmark (N=3,862)	**-0.15**	-0.07	0.06	**0.14**
	**(-0.29, -0.02)**	(-0.23, 0.08)	(-0.06, 0.17)	**(0.01, 0.28)**
England (N=6,657)	**-0.05**	0.03	**0.09**	**0.06**
	**(-0.10, -0.00)**	(-0.01, 0.07)	**(0.04, 0.14)**	**(0.02, 0.10)**
Estonia (N=6,657)	**-0.14**	0.03	**0.13**	**0.12**
	**(-0.20, -0.07)**	(-0.02, 0.07)	**(0.04, 0.21)**	**(0.03, 0.20)**
France (N=5,576)	**-0.17**	0.04	0.02	**0.13**
	**(-0.23, -0.10)**	(-0.02, 0.09)	(-0.05, 0.10)	**(0.07, 0.19)**
Germany (N=5,212)	-0.08	0.05	0.03	0.04
	(-0.18, 0.02)	(-0.04, 0.14)	(-0.09, 0.14)	(-0.06, 0.14)
Italy (N=5,933)	**-0.07**	0.03	**0.17**	**0.06**
	**(-0.13, -0.02)**	(-0.01, 0.08)	**(0.11, 0.23)**	**(0.01, 0.11)**
Slovenia (N=4,616)	**-0.08**	**0.07**	0.02	**0.14**
	**(-0.14, -0.01)**	**(0.004, 0.13)**	(-0.06, 0.10)	**(0.07, 0.21)**
Spain (N=6,694)	**-0.10**	**0.07**	-0.02	**0.15**
	**(-0.15, -0.05)**	**(0.03, 0.11)**	(-0.08, 0.04)	**(0.10, 0.20)**
Sweden (N=4,822)	**-0.20**	**0.18**	**0.27**	**0.14**
	**(-0.32, -0.07)**	**(0.04, 0.32)**	**(0.12, 0.43)**	**(0.02, 0.26)**
Switzerland (N=3,487)	**0.04**	**0.20**	**-0.04**	**0.16**
	**(-0.05, 0.14)**	**(0.11, 0.29)**	**(-0.15, 0.08)**	**(0.07, 0.24)**
United States (N=10,746)	**-0.10**	**0.04**	**0.09**	**0.09**
	**(-0.13, -0.06)**	**(0.01, 0.06)**	**(0.06, 0.13)**	**(0.06, 0.12)**

Note: The models controlled for all time-constant variables and time-varying variables including age, partnership status, number of people living in the household, employment status, household income, housing tenure, long-standing mental/physical conditions, difficulties with daily activities (ADLs), and difficulties with instrumental activities of daily living (IADLs). Bold values denote statistical significance at the $p<0.05$ level.

Supplementary Table 4 | Subgroup analyses by hobbies measures in meta-analysis

	Effect size	95%CI	I^2	τ^2	H^2	$P_{subgroup}$
Depressive symptoms						
Hobbies measures						0.645
Listed	-0.10	-0.15, -0.06	61.6%	0.004	2.60	
Binary	-0.09	-0.14, -0.03	83.8%	0.002	6.17	
Self-reported health						
Hobbies measures						0.818
Listed	0.06	0.03, 0.09	32.3%	0.001	1.48	
Binary	0.05	0.00, 0.10	81.8%	0.002	5.50	
Happiness						
Hobbies measures						0.578
Listed	0.09	0.04, 0.14	61.3%	0.005	2.58	
Binary	0.10	0.08, 0.12	0.00%	0.000	1.00	
Life satisfaction						
Hobbies measures						0.433
Listed	0.11	0.07, 0.15	49.0%	0.002	1.96	
Binary	0.09	0.07, 0.11	0.23%	0.000	1.00	

Note: N study = 16. I^2 indicates the percentage of variability in the effect size that is caused by between-study heterogeneity, rather than by sampling error. τ^2 indicates the variance of the true effect sizes underlying our data. H^2 statistics describe the ratio of the observed variation and the expected variance due to sampling error. A value of H^2 greater than 1 indicates the presence of between-study heterogeneity. $P_{subgroup}$ presents the p-value of the test for subgroup differences to test whether the difference between subgroups is statistically significant.

Supplementary Table 5 | Hobbies and mental wellbeing measures across five longitudinal studies

Data	Survey questions
Hobby	
ELSA	'I have a hobby or past-time' [1=yes; 0=no]
JAGES	'Do you have hobbies?' [1=yes; 0=no]
HRS	'How often do you work on a hobby or project?' [1=any frequency; 0=not in the last month/never]
SHARE	'Which of the activities listed on this card – if any – have you done in the past twelve months?' **1.** Done voluntary or charity work. **2.** Attended an educational or training course. **3.** Gone to a sport, social or other kind of club. **4.** Taken part in a political or community-related organization. **5.** Read books, magazines or newspapers. **6.** Did word or number games such as crossword puzzles or Sudoku. **7.** Played cards or games such as chess. **0.** None of these [1=any of the activities; 0=none]
CHARLS	'Which of the social activities listed on this card – if any – have you done in the past month?' **1.** Played ma-jong, played chess, played cards or went to a community club. **2.** Went to a sport, social or other kind of club. **3.** Took part in a community-related organisation. **4.** Did voluntary or charity work. **5.** Attended an educational or training course. **0.** None of these [1=any of the activities; 0=none]
Depressive symptoms	
ELSA	Center for Epidemiologic Studies Depression Scale (CES-D) [8 items]
JAGES	Geriatric Depression Scale (GDS) [15 items]
HRS	CES-D [8 items]
SHARE	EURO-D scale [12 items]
CHARLS	CED-D [10 items]
Self-reported health	
ELSA	'Would you say your health is excellent, very good, good, fair, or poor?' [reversely coded]
JAGES	'How is your health condition now?' – ranging in excellent, good, fair, or poor [reversely coded]
HRS	'Would you say your health is excellent, very good, good, fair, or poor?' [reversely coded]
SHARE	'Would you say your health is excellent, very good, good, fair, or poor?' [reversely coded]
CHARLS	'Would you say your health is very good, good, fair, poor or very poor?' [reversely coded]
Happiness	
ELSA	'Overall, how happy did you feel yesterday?' [11-point scale]
JAGES	'How happy are you now?' [11-point scale]
HRS	'During the past 30 days, to what degree did you feel happy?' [5-point scale]
SHARE	'How often, on balance, do you look back on your life with a sense of happiness?' [4-point scale]
CHARLS	'How have you felt and behaved during the last week? - I was happy.' [4-point scale]
Life satisfaction	
ELSA	'I am satisfied with my life' [7-point scale]
JAGES	'Are you satisfised with your present life?' [1=yes; 0=no]
HRS	'I am satisfied with my life.' [7-point scale]
SHARE	'How satisfied are you with your life?' [10-point scale]
CHARLS	How satisfied are you with it? [5-point scale]

Note: ELSA refers to the English Longitudinal Study of Ageing. JAGES refers to the Japan Gerontological Evaluation Study. HRS refers to the Health and Retirement Study. SHARE refers to the Survey of Health, Ageing and Retirement in Europe. CHARLS refers to the China Health and Retirement Longitudinal Study.
