# Supplementary material for: Hobby engagement and mental wellbeing among people aged 65 years and older in 16 countries
Source: Nat Med. 2023 Sep 11;29(9):2233–40. doi: 10.1038/s41591-023-02506-1 (PMC10504079; doi:10.1038/s41591-023-02506-1)
Supplement: Supplementary file 2 — Reporting Summary [file 41591_2023_2506_MOESM2_ESM.pdf]

nature portfolio

Corresponding author(s): Daisy Fancourt

Last updated by author(s): Jul 5, 2023

Reporting Summary

Nature Portfolio wishes to improve the reproducibility of the work that we publish. This form provides structure for consistency and transparency in reporting. For further information on Nature Portfolio policies, see our Editorial Policies and the Editorial Policy Checklist.

Statistics

For all statistical analyses, confirm that the following items are present in the figure legend, table legend, main text, or Methods section.

n/a	Confirmed	
☐	☒	The exact sample size (*n*) for each experimental group/condition, given as a discrete number and unit of measurement
☐	☒	A statement on whether measurements were taken from distinct samples or whether the same sample was measured repeatedly
☐	☒	The statistical test(s) used AND whether they are one- or two-sided<br>*Only common tests should be described solely by name; describe more complex techniques in the Methods section.*
☐	☒	A description of all covariates tested
☒	☐	A description of any assumptions or corrections, such as tests of normality and adjustment for multiple comparisons
☐	☒	A full description of the statistical parameters including central tendency (e.g. means) or other basic estimates (e.g. regression coefficient) AND variation (e.g. standard deviation) or associated estimates of uncertainty (e.g. confidence intervals)
☐	☒	For null hypothesis testing, the test statistic (e.g. *F*, *t*, *r*) with confidence intervals, effect sizes, degrees of freedom and *P* value noted<br>*Give P values as exact values whenever suitable.*
☒	☐	For Bayesian analysis, information on the choice of priors and Markov chain Monte Carlo settings
☐	☒	For hierarchical and complex designs, identification of the appropriate level for tests and full reporting of outcomes
☐	☒	Estimates of effect sizes (e.g. Cohen's *d*, Pearson's *r*), indicating how they were calculated

Our web collection on statistics for biologists contains articles on many of the points above.

Software and code

Policy information about availability of computer code

Data collection	No software was used for data collection.
Data analysis	Fixed effects regression; Meta-analyses using the random effect model; Meta-analyses for subgroup differences; Meta regression; Ordinary Least Squares regressions; Correlation test; Arellano-Bond estimator analyses; Multi-level analyses; Multiple imputation by chained equations. Stata v17 was used for the analyses. All code used for these analyses is publicly available online: https://osf.io/84xzu/

For manuscripts utilizing custom algorithms or software that are central to the research but not yet described in published literature, software must be made available to editors and reviewers. We strongly encourage code deposition in a community repository (e.g. GitHub). See the Nature Portfolio guidelines for submitting code & software for further information.

Data

Policy information about availability of data

All manuscripts must include a data availability statement. This statement should provide the following information, where applicable:

- Accession codes, unique identifiers, or web links for publicly available datasets
- A description of any restrictions on data availability
- For clinical datasets or third party data, please ensure that the statement adheres to our policy

The English Longitudinal Study of Ageing (ELSA) can be accessed via the UK Data Service: https://beta.ukdataservice.ac.uk/datacatalogue/series/series?id=200011
The Health and Retirement Study (HRS) can be accessed via the RAND Center for the Study of Aging: https://hrsdata.isr.umich.edu/data-products/rand

The Survey of Health, Ageing and Retirement in Europe (SHARE) can be accessed via the SHARE Research Data Center: http://www.share-project.org/data-access.html
The China Health and Retirement Longitudinal Study (CHARLS) can be accessed via the National School of Development, Peking University: https://charls.charlsdata.com/pages/data/111/en.html
Restrictions to access data of Japan Gerontological Evaluation Study (JAGES) applied. For researchers who wish to use the data, please contact the JAGES Data Administration Office at dataadmin.ml@jages.net. Non-JAGES research members may be required to include JAGES members in their project or co-authors in research papers depending on the study topic or data used.
All code used for these analyses is publicly available online: https://osf.io/84xzu/

Human research participants

Policy information about studies involving human research participants and Sex and Gender in Research.

Reporting on sex and gender	The gender information was collected in all five longitudinal studies analysed in this study. The distribution of gender is presented in Table 1. As this study used the fixed-effects modelling approach, gender and other time-invariant variables have already been automatically controlled. The main analyses apply to the whole sample, including both genders, and additional analyses stratifying by gender are presented in Extended Data Figures 8-9.
Population characteristics	The average age of the respondents across the different countries was between 71.7 and 75.9 years. Generally, there was a higher proportion of females participating in the surveys (except for China, Japan, and Germany). More than seven out of ten were retired, except for those living in China, Japan, and Spain. Over 60% of the participants experienced long-standing mental or physical health conditions. For hobby engagement, Denmark (96.0%), Sweden (95.8%) and Switzerland (94.4%) had the highest engagement levels, followed by Germany (91.0%), Austria (90.0%) and Japan (90.0%). Spain (51.0%), Italy (54.0%) and China (37.6%; albeit focusing exclusively on social hobbies) had the lowest engagement levels. Full description of the population characteristics is reported in Table 1.
Recruitment	Given that the study analysed quantitative, secondary datasets, authors were not involved in data collection or recruitment. We have however provided data availability resources in the manuscript which provide full information on data collection.
Ethics oversight	Each analytical dataset provided guidelines for study procedures. For ELSA, Wave 9 received ethical approval from the South Central – Berkshire Research Ethics Committee on 10th May 2018 (17/SC/0588). Wave 8 received ethical approval from the South Central – Berkshire Research Ethics Committee on 23rd September 2015 (15/SC/0526). Wave 7 received ethical approval from the NRES Committee South Central - Berkshire on 28th November 2013 (13/SC/0532). Wave 6 received ethical approval from the NRES Committee South Central - Berkshire on 28th November 2012 (11/SC/0374). Wave 5 received ethical approval from the Berkshire Research Ethics Committee on 21st December 2009 (09/H0505/124). Wave 4 received ethical approval from the National Hospital for Neurology and Neurosurgery & Institute of Neurology Joint Research Ethics Committee on 12th October 2007 (07/H0716/48). Wave 3 received ethical approval from the London Multi-Centre Research Ethics Committee on 27th October 2005 (05/MRE02/63). Wave 2 received ethical approval from the London Multi-Centre Research Ethics Committee on 12th August 2004 (MREC/04/2/006). Wave 1 received ethical approval from the London Multi-Centre Research Ethics Committee on 7th February 2002 (MREC/01/2/91). All participants provided informed written consent.<br><br>For JAGES, the study received ethical approval from Nihon Fukushi University (No. 10-05), Chiba University (No. 2493) and the National Center for Geriatrics and Gerontology (No. 992), and all participants provided informed written consent.<br><br>For HRS, ethical approval for HRS was obtained from the University of Michigan Institutional Review Board. All participants gave informed written consent.<br><br>For SHARE, the study received ethical approval from the Ethics Council of the Max Planck Society and all participants provided informed written consent.<br><br>For CHARLS, the study received ethical approval from the Biomedical Ethics Review Committee of Peking University (IRB00001052-11015), and all participants provided informed written consent.

Note that full information on the approval of the study protocol must also be provided in the manuscript.

Field-specific reporting

Please select the one below that is the best fit for your research. If you are not sure, read the appropriate sections before making your selection.

☐ Life sciences ☒ Behavioural & social sciences ☐ Ecological, evolutionary & environmental sciences

For a reference copy of the document with all sections, see nature.com/documents/nr-reporting-summary-flat.pdf

Behavioural & social sciences study design

All studies must disclose on these points even when the disclosure is negative.

Study description	This study harmonised quantitative measures of hobby engagement and multiple aspects of psychological health across 16 nations represented in five longitudinal studies. Fixed effects models and multi-national meta-analyses were applied to compare the longitudinal associations between hobbies and mental health internationally.
Research sample	Participants were aged 65 and above and were involved in one of the five longitudinal studies across 16 countries: English Longitudinal Study of Ageing (ELSA), Japan Gerontological Evaluation Study (JAGES), US Health and Retirement Study (HRS), Survey of Health, Ageing and Retirement in Europe (SHARE), and China Health and Retirement Longitudinal Study (CHARLS).<br><br>ELSA started in 2002/03 and follows over 11,000 participants aged 50 years+ living in England every two years.<br><br>JAGES is a large-scale population-based longitudinal study about ageing established in 2010, mainly collected through self-administered mail surveys, targeting older people aged 65+ who do not receive long-term care insurance benefits. ELSA Waves 7-9 were analysed. JAGES has conducted joint survey with municipalities that are the public insurers of long-term care insurance every 3-4 years: Wave 1 (2010/11) to Wave 4 (2019/2020). JAGES Waves 2-4 were analysed.<br><br>HRS is a national cohort study of more than 37,000 individuals over the age of 50 in the US. The study was initiated by the National Institute on Aging and conducted by the Institute for Social Research at the University of Michigan. The initial HRS cohort was interviewed for the first time in 1992 and followed-up every two years, with other studies and younger cohorts merged with the initial pool of respondents. Together, these studies create a group of fully representative respondents aged over 50 in the United States. HRS Waves 9-14 were analysed, where at each wave, a rotating random 50% sub-group of respondents were invited to an enhanced interview and given a Leave-Behind Psychosocial and Lifestyle Questionnaire to complete and return by mail.<br><br>SHARE is the largest pan-European social science panel study providing internationally comparable longitudinal micro data on the population aged 50+ and currently includes eight waves with data collection starting in 2004. SHARE contains both the participation of respondents in their baseline and refreshment interview to account for a reduction in the number of respondents due to panel attrition. SHARE Waves 4, 5 and 6 were analysed.<br><br>CHARLS is a national cohort study of Chinese residents aged 45+. The baseline survey started in 2011 and has been followed up every two years (in 2013 and 2015). Multistage probability sampling was used for a selection of respondents. CHARLS Waves 1-3 were analysed.<br><br>The proportion of gender for each country is reported in Table 1. The sample is not representative but heterogenous which involves a wide pool of respondents from different socio-demographic backgrounds and with various health conditions.
Sampling strategy	The sample strategy varies across the five datasets. More information can be found in the data resources. This study only considered respondents aged 65 years+ to allow for comparison across all datasets. The sample is sufficient as the age group reflects the ageing population.
Data collection	No data collection was performed for this study as existing secondary datasets were used for analyses. Data collection procedure for each longitudinal study can be found in the data resources.
Timing	ELSA started in 2002/03. JAGES started in 2010/11. HRS started in 1992. SHARE started in 2004. CHARLS started in 2011. Intervals between interview waves vary by studies. All five longitudinal studies are still on-going.
Data exclusions	ELSA, HRS, SHARE and CHARLS excluded data where participants were aged under 65. All longitudinal studies excluded data where participants did not respond to all measures.
Non-participation	The proportion of missingness was as follows: Austria (20.6%), Belgium (39.6%), China (69.7%; mainly due to missingness in household wealth, hobby and life satisfaction), Czech Republic (34.8%), Denmark (56.6%), England (15.1%), Estonia (20.8%), France (21.2%), Germany (69.1%), Italy (51.3%), Japan (54.0%), Slovenia (54.8%), Spain (53.8%), Sweden (62.5%), Switzerland (19.3%), and the US (15.7%). The missingness was largely due to non-response and attrition. To replicate the analysis on a complete data, we additionally performed multiple imputations as a sensitivity analysis. Results were largely replicated and are reported in the manuscript.
Randomization	Not applicable as longitudinal studies follow a group of participants over a period of time. However, the main analyses control for all time-invariant variables and important time-varying variables, including age, partnership status, number of people living in the household, employment status, household income, housing tenure, long-standing mental/physical conditions, difficulties with daily activities and difficulties with instrumental activities of daily living.

Reporting for specific materials, systems and methods

We require information from authors about some types of materials, experimental systems and methods used in many studies. Here, indicate whether each material, system or method listed is relevant to your study. If you are not sure if a list item applies to your research, read the appropriate section before selecting a response.

Materials & experimental systems

n/a	Involved in the study
☒	☐ Antibodies
☒	☐ Eukaryotic cell lines
☒	☐ Palaeontology and archaeology
☒	☐ Animals and other organisms
☒	☐ Clinical data
☒	☐ Dual use research of concern

Methods

n/a	Involved in the study
☒	☐ ChIP-seq
☒	☐ Flow cytometry
☒	☐ MRI-based neuroimaging
